# Relationship between Repeated Sprint Ability and Aerobic Capacity in Professional Soccer Players

**DOI:** 10.1155/2013/952350

**Published:** 2013-10-01

**Authors:** Rhys M. Jones, Christian C. Cook, Liam P. Kilduff, Zoran Milanović, Nic James, Goran Sporiš, Bruno Fiorentini, Fredi Fiorentini, Anthony Turner, Goran Vučković

**Affiliations:** ^1^Sport and Exercise Science Research Centre, Talbot Building, Swansea University, Swansea SA2 8PP, UK; ^2^UK Sport Council, 40 Bernard Street, London, UK; ^3^Faculty of Sport and Physical Education, University of Nis, Čarnojevićeva 10a, 18000 Nis, Serbia; ^4^London Sport Institute, Middlesex University, London NW4 4BT, UK; ^5^Faculty of Kinesiology, University of Zagreb, 10000 Zagreb, Croatia; ^6^Ken Blanchard College of Business, Grand Canyon University, AZ 85017, USA; ^7^Faculty of Kinesiology, University of Split, 21000 Split, Croatia; ^8^Faculty of Sport, University of Ljubljana, 1000 Ljubljana, Slovenia

## Abstract

*Aim*. The aim of the present study was to investigate the relationship between maximal aerobic capacity (VO_2 max_) and repeated sprint ability (RSA) in a group of professional soccer players. *Methods*. Forty-one professional soccer players (age 23 ± 4 yrs, height 180.0 ± 5.3 cm, weight 79.6 ± 5.3 kg) were required to perform tests to assess RSA and VO_2 max_ on two separate days with at least 48 hr rest between testing sessions. Each player performed a treadmill test to determine their VO_2 max_ and a test for RSA involving the players completing 6 × 40 m sprints (turn after 20 m) with 20 s active recovery between each sprint. *Results*. There was a significant negative correlation between body mass normalised VO_2 max_ and mean sprint time (RSA_mean_) (*r* = −0.655; *P* < 0.01) and total sprint time (RSA_total_) (*r* = −0.591, *P* < 0.01). *Conclusion*. Results of the current study indicate that VO_2 max_ is one important factor aiding soccer players in the recovery from repeated sprint type activities.

## 1. Introduction

The ability to perform (and recover from) repeated high intensity activities over a prolonged period of time coupled with a good aerobic capacity is deemed essential physiological requirements for success in soccer [1]. For example, maximal aerobic capacity (VO_2max⁡_) in soccer squads has been correlated to playing standard [2] and league position [3], suggesting that higher VO_2max⁡_ values enable players to have a physiological advantage to perform in elite soccer. Further support of this fact can be taken from the work of Rampinini et al. [4] who reported significant correlations between peak speed reached during an incremental field test and total distance covered (*r* = 0.58), high intensity running (*r* = 0.65), and very high intensity running during competitive games (*r* = 0.64).

In addition to the importance of having a good aerobic capacity, players also perform approximately 150–250 brief intense bouts of activity during a game with these bouts of activity contributing to the more crucial moments of the game such as winning possession, shooting, and chasing a player to tackle [5]. Using match analysis technology, it has been demonstrated that international players perform 28% more high intensity running and 58% more sprinting than professional players of a lower standard [5]. The amount of high intensity exercise performed (and recovery from it) is one factor that helps distinguish top-class players from players of a lower standard [1]. Players who can regularly perform repeat sprint efforts at the same or very similar intensity and quality are therefore likely to perform better over extended periods of time [4]. The extent to which an individual can maintain their sprint performance is known as “repeated sprint ability” (RSA) [6] which is largely dependent on the extent of Phosphocreatine (PCr) resynthesis [7] and the removal of hydrogen ions (H^+^) from the muscle during recovery between bouts. It has previously been proposed that an individual's RSA may be aided by their aerobic capacity [8] as an enhanced aerobic capacity may increase the ability to tolerate, remove, and buffer H^+^ from the working muscle [9] and also enhance PCr and adenosine triphosphate (ATP) resynthesis from inorganic phosphates postexercise [10]. 

To date, only a limited number of studies have examined the relationship between VO_2max⁡_ and RSA with one study reporting a significant relationship between VO_2max⁡_ and total sprint time (RSA_total_; *r* = −0.49) in a group of amateur team sport and racquet athletes [6]. Additional research examining the existence of a relationship between RSA and VO_2max⁡_ in elite athletes has produced equivocal findings [8, 11–14]. To the authors knowledge, only one paper has found a relationship between RSA and VO_2max⁡_ using elite soccer players [8]. However, a major limitation of this work and other research which has found no significant relationship between RSA and VO_2max⁡_ is the lack of reliability and logical, construct, and criterion validity of RSA protocols in reference to the sport in question. Furthermore, studies have indirectly determined VO_2max⁡_ using field tests [11, 14] which is likely to result in up to 10 ± 15% inaccuracy level [15]. Recently, a protocol to assess RSA specific to the demands of elite soccer has been implemented which has demonstrated reliability and validity [4, 16]. The repeated sprint protocol involves six 40 m shuttle sprints separated by 20 s passive recovery, with each sprint including a 180° turn after 20 m, thus including a deceleration, turn, and acceleration, specific to the demands of soccer. Furthermore, mean repeated sprint time (RSA_mean_) has been reported as 7.25 ± 0.17 s (range 6.95–7.50 s) in elite soccer players; thus work to rest ratios of 1 : 2.8 are achieved with the mean total duration lasting 143.5 s, which is similar to the most demanding phases of a soccer match giving the protocol logical validity [4]. 

In light of the above the present study aimed to determine whether a relationship exists between RSA and VO_2max⁡_ in professional soccer players. We hypothesized that VO_2max⁡_ would be associated with RSA in professional soccer players.

## 2. Methods

### 2.1. Subjects

Forty-one professional soccer players (age 23 ± 4 yrs, height 180.0 ± 5.3 cm, weight 79.6 ± 5.3 kg), from whom written informed consent had been obtained, volunteered to take part in the present study which was approved by the universities ethics committee. Players refrained from heavy training for the 2 days prior to testing. During the 2 hours before testing they were only allowed ad libitum water intake with subjects consuming a light meal 3 hours before testing. Players were accustomed to the testing procedures and had performed VO_2max⁡_ and tests for RSA on a number of occasions prior to the commencement of the present study. The present study was performed at the end of preseason training for the season beginning 2008-2009.

### 2.2. Experimental Procedures

For both tests, players were asked to refrain from alcohol and caffeine 24 hrs prior to testing reducing the possibility of dehydration affecting performance. During all testing players were given encouragement from testing and club staff.

### 2.3. Measurements

#### 2.3.1. Maximal Aerobic Uptake (VO_2max_)

VO_2max⁡_ was determined following an incremental treadmill run to fatigue (Woodway Ergo ELG 55 treadmill; Woodway GmbH, Weil am Rhein, Germany) with gas exchange and ventilatory variables being analysed breath-by-breath using a calibrated computer-based exercise system (Jaeger Oxycon Pro online gas analyser; Erich Jaeger GmbH, Hoechberg, Germany). The CO_2_ and O_2_ analyzers were calibrated before each test using a two-point measure: a calibration gas (CO_2_ 5%, O_2_ 16%, N_2_ balance) and a reference gas (room air after ATPS (ambient temperature and pressure, saturated) to STPD (standard temperature and pressure, dry)). Heart rate (HR) was continuously recorded (Polar; Lake Success, NY, USA). 

Following the measurement of each subject's stature (Holtain Stadiometer; Holtain Ltd., Crymych, Wales) and body mass (Seca 888 Class III floor scale; Seca, Birmingham, UK) subjects performed 5 min light running at a self-selected pace. The protocol for the exercise test was preinstalled to allow automatic and accurate increments in speed (km·h^−1^) and gradient (%) (Table 1). 

#### 2.3.2. Repeated Sprint Ability (RSA)

The RSA protocol required each subject to complete six 40 m sprints with 20 s active recovery between each sprint. A sprint consisted of the subject taking up a start position with their leading foot placed 30 cm behind the start line, which was instrumented with timing gates (Brower timing systems; Draper, UT, USA). When ready the subject sprinted through the timing gates (starting the timing) to a line marked on the track 20 m from the start line. Once the subject's foot touched on the line at the 20 m mark, they turned (as fast as possible) and sprinted back. Timing stopped when the subject recrossed the start line.

Prior to testing, subjects performed a 10 min warm-up which included light running and dynamic stretching, followed by several maximal sprints over 20 m. Three minutes following the warm-up, players underwent 2 preliminary 40 m shuttle sprints, with the fastest time used as a criterion score for the repeated sprint test. Five minutes from the second criterion run, subjects began the RSA test. If the first sprint during the RSA test 2.5% or more slow than the criterion time, then the repeated sprint performance was deemed not to be maximal and discarded from analysis. Performance variables analysed from the RSA test were total sprint time (RSA_total_) (time for all 6 sprints combined) and mean sprint time (RSA_mean_) (average over the 6 sprints). 

The reliability (typical error expressed as a coefficient of variation) for RSA_mean_ has been reported to be 0.8% [17]. In addition the validity of the test was assessed and significant correlations between RSA_mean_ time and match performance variables (i.e., very high intensity running and sprinting distance) as assessed match analysis systems [4].

### 2.4. Statistical Analysis

Data are presented as the mean ± standard deviation (SD). A two-tailed Pearson's product-moment correlation was used to determine the strength and directionality of the relationship between VO_2max⁡_ and RSA (RSA_mean_, RSA_total_). The coefficient of determination (*r* ^2^) was used to examine the amount of explained variance between tests. The level of significance was set at *P* < 0.05. 

## 3. Results

**Figure 1:**
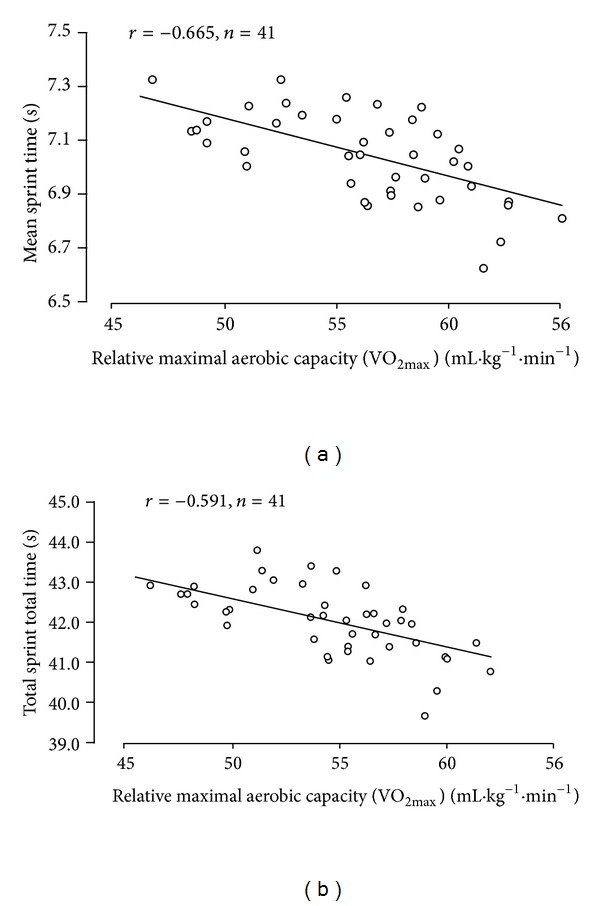
Relationship between relative aerobic capacity and mean sprint time (a) and total sprint time (b).

The correlation coefficients between absolute and relative VO_2max⁡_ and the RSA performance indicators are summarised in Table 2. There were significant negative correlations between relative VO_2max⁡_ and RSA_mean_ (*r* = −0.655, *P* < 0.01; Figure 1(a)) and RSA_total_ (*r* = −0.591, *P* < 0.01; Figure 1(b)). However, no such correlation existed between absolute VO_2max⁡_ and RSA_mean_ or RSA_total_ (Table 2). 

## 4. Discussion

The present study examined the relationships between aerobic capacity and repeated sprint ability in a group of professional soccer players. The key finding from the present study was that significant moderate negative correlations were found between VO_2max⁡_ (mL·kg·min^−1^) and RSA expressed as RSA_mean_ (*r* = −0.655, *P* < 0.01) and RSA_total_ (*r* = −0.591, *P* < 0.01). The present study supports the theory that aerobic capacity is an important factor influencing recovery from RSA in elite soccer. The ability of an enhanced VO_2max⁡_ to improve recovery between repeated bouts of maximal sprinting is likely related to the ability to tolerate, remove, and buffer hydrogen ions (H^+^) from the working muscle [9] while efficiently restoring PCr and ATP stores from inorganic phosphates postexercise [7]. Previous research has shown that the extent of PCr degradation and H^+^ accumulation, which increases with repeated bouts of maximal exercise [18], is associated with muscular fatigue in soccer players. Various mechanisms could be proposed; for example, an individual with a higher VO_2max⁡_ may exhibit an increased mitochondrial number, size, and surface area [19] which may allow greater movement of pyruvate into the mitochondria. Increased concentrations of aerobic enzymes will enhance the capacity to generate ATP by oxidative phosphorylation. Furthermore, individuals with an increased VO_2max⁡_ may have increased myoglobin concentrations [20]. This would enhance the ability of the skeletal muscle to move oxygen from the muscle cell membrane to the mitochondria and will increase the magnitude of myoglobin oxygen stores, thus increasing the delivery of oxygen (O_2_) to the mitochondria at the onset of exercise. Furthermore, an elevated VO_2max⁡_ has been proposed to directly enhance lactate removal and PCr restoration, thus enhancing power and force recovery. Lactate removal is dependent on a series of events with approximately 65% of the lactate converted to pyruvate by lactate dehydrogenase (LDH), which then undergoes aerobic degradation via the krebs cycle and electron transport system with the nonconverted 35% secreted as urine and sweat or converted to protein [21]. If an enhanced VO_2max⁡_ is associated with enhanced aerobic enzyme concentrations and an increased mitochondrial surface area providing greater transport sites for pyruvate [19], it is likely to enhance lactate removal. 

Previous research supporting the relationship between VO_2max⁡_ and RSA has been limited in elite athletes. For a study to assess RSA for a specific sport, the protocol used should be specific to the physiological response and activity pattern of the most intense repeated sprint activity observed from the sport (logical validity), should be reliable, and should have construct and criterion validity [11]. Studies that have utilised indirect methods of determining VO_2max⁡_ [11, 14] including the multistage fitness test which is likely to have 10–15% inaccuracy are therefore inaccurate when assessing the relationship between RSA and VO_2max⁡_. 

In contrast with the results of this study, some studies [8, 22] have reported that VO_2max⁡_ is a poor indicator of RSA (*r* = 0.09–0.03). This may be explained by differences in the type of protocol used for the RSA test [23] where VO_2max⁡_ has not been reported to be related to RSA when sprints of less than 40 meters or 6 seconds have been used. Furthermore, RSA may also be influenced by the training status of the subjects [24]. Da Silva et al. [23] have concluded that the minimum velocity needed to reach VO_2max⁡_ and velocity at the onset of blood-lactate accumulation were more strongly correlated with RSA indices than VO_2max⁡_.

A key physiological requirement of most intermittent team sports is the ability to repeatedly produce short, maximal effort activities with incomplete (and varying) recovery (repeated sprint ability (RSA)). Soccer players are required to frequently produce brief intense actions (150–200 per game) [5] which constitute some of the most crucial moments of the game [1], and time motion analysis has demonstrated that players in all playing positions experience a significant decline in the number of high intensity actions performed across a match [5]. Furthermore, the ability to limit this decline in high intensity activities appears to be associated with playing standard with international players performing 28% more high intensity running and 58% more sprinting than professional players of a lower standard [5]. This suggests the ability to recover between bouts of high intensity activity may be a key determinant of performance in elite soccer. However, the physiological mechanism facilitating recovery between repeated sprints has previously been unclear. With aerobic contribution increasing with the number of repeated bouts and oxygen uptake potential contributing to recovery between maximal bouts of sprinting, it has previously been proposed that an enhanced aerobic capacity may influence recovery between bouts of high intensity activity [8]. Further support for this theory has come from match analysis data in elite soccer which has shown that improvements in VO_2max⁡_ (58.1 ± 4.5 mL·kg·min^−1^ to 64.3 ± 3.9 mL·kg·min^−1^) following 8 weeks of interval training lead to significant improvements in distance covered during games (20%), number of sprints (100%), number of involvements with the ball (24%), and work intensity measured as percentage of maximal heart rate (82.7 ± 3.4% to 85.6 ± 3.1% [25]. Furthermore, players with greater VO_2max⁡_ values have been found to cover greater total distances and distances at high intensity during a match [25]. Thus match analysis data suggests that an improved VO_2max⁡_ gives an enhanced potential to recover between bouts of maximal intensity activity allowing greater distances at a higher intensity to be covered during a match.

To the authors knowledge, the present study is the first to assess the relationship between RSA and VO_2max⁡_ using an RSA protocol shown to be reliable and valid, while determining VO_2max⁡_ directly from breath-by-breath analysis taken during an incremental treadmill run to fatigue. As a consequence the finding from the present study is to date the most conclusive evidence in the assessment of the relationship between RSA and VO_2max⁡_ in elite athletes. 

A limitation of this study is that the minimum velocity needed to reach VO_2max⁡_ and velocity at the onset of blood-lactate accumulation were not assessed. Also, we did not assess anaerobic capacity and its relationship with repeated sprint ability in professional soccer players. Future study is required to document relationship between aerobic (velocity needed to reach VO_2max⁡_ and velocity at the onset of blood-lactate accumulation) and anaerobic capacity at the one side and RSA at the other side and determine which of them are better predictor of RSA in professional soccer players.

In conclusion, the results from the present study suggest that, in professional soccer players, there is a relationship between RSA and relative VO_2max⁡_. This suggests that, to improve RSA, it is important to implement specific soccer training targeting aerobic components.

## Figures and Tables

**Table 1:** Incremental treadmill test protocol.

Time (min)	Speed (km·h^−1^)	Gradient (%)
0–2	10	0
2–4	12	0
4–6	14	+2.5
6–8	16	+5.0
8–10	18	+5.0
10–12	20	+5.0

**Table 2:** Correlation coefficients between maximal oxygen uptake and repeated sprint ability test performance indices.

Repeated sprint ability	Maximal oxygen uptake	
	Relative	Absolute
Mean sprint time (s)	−0.655*	−0.068
Total sprint time (s)	−0.591*	0.033

*indicates *P* < 0.05.
